# Seven incidents within a fortnight in Nepal: Is violence against healthcare professionals curbed by tougher laws?

**DOI:** 10.1016/j.dialog.2024.100173

**Published:** 2024-02-19

**Authors:** Leison Maharjan

**Affiliations:** Patan Academy of Health Sciences, Lalitpur, Nepal; Department of Otolaryngology and Head & Neck Surgery, Patan Academy of Health Sciences, Lalitpur, Bagmati Province, Nepal

**Keywords:** Assault, Health professionals, Patient aggression, Physical abuse, PHYSICIANS, Violence, Workplace violence

## Abstract

In September 2023, a surge of violence against healthcare professionals occurred in Nepal within a two-week span, despite recent legal amendments aimed at curbing such incidents. This manuscript explores whether stricter legislation effectively deters these acts. The violence is rooted in Nepal's healthcare system's inadequacies, leading to overcrowded and understaffed hospitals, patient frustration, and healthcare professional burnout. Misinformation and rumors, particularly in rural areas, can trigger outbreaks of violence, exacerbated by media sensationalism. The lack of legal consequences for attackers is a significant factor. Perpetrators often go unpunished, emboldening others to resort to violence when dissatisfied with medical services. Political affiliations and third-party involvement for financial gain are common. The psychological toll on healthcare workers is profound, resulting in burnout, depression, and post-traumatic stress disorder, contributing to a significant brain drain of doctors from Nepal. This paper underscores the importance of enforcing existing laws to create a safe workplace and making the malpractice complaint process accessible to the public to deter resorting to violence.

In September 2023, a surge of violence was observed within a matter of two weeks in Nepal. The incident was even addressed by the president of the World Medical Association (WMA) at the 74^th^ General Assembly in Kigali, Rwanda on October 7, 2023 [1].

On September 13 (Sancho Hospital, Hetauda), two consultants were assaulted following the death of a patient from dengue. One of them even sustained a zygomatic fracture [2]. On September 14 (Birat Medical College, Biratnagar), the hospital was vandalized following the death of the patient. On September 21 (RK Medicity Hospital, Siraha), a radiographer was beaten following a case of motorbike accident accusing him that he did not perform a quick Xray [ 3]. On September 24 (Lamjung Hospital, Lamjung), a medical officer was violently assaulted when he asked for a written consent before admitting a patient suffering from dengue [4]. On September 25 (Manipal College of Medical Sciences, Pokhara), a resident doctor got beaten after a patient with chronic kidney disease who was being treated on a ventilator died. Dozens of local people gathered and vandalized the hospital [ 5]. On September 25 (Patan Hospital, Lalitpur), a medical officer was assaulted after a patient with lung cancer under palliative care died [ 6]. On September 26 (Baghauda Hospital, Chitwan), a medical officer was beaten while he was performing CPR [ 7].

In response to this alarming trend, healthcare workers in Nepal, under the leadership of the Nepal Medical Association (NMA), initiated a strike demanding legal protection. It was supported not only by the doctors but also by other paramedical staffs. All the health workers withdrew from the routine work except the emergency part. The strike ended with a negotiation with the Ministry of Health and Population [ 8]. Not to forget that merely a year ago, a First Amendment 2079 B.S. (2022 A.D.) was made through the Ordinance by the President of Nepal, adding a harsher penalty in the “Security of Health Workers and Health Institutions Act, 2066 B.S.” (2010 A.D.) with a fine up to Rs 300,000 and/or jail term up to 3 years depending upon the nature of violence against health workers [9,10]. Compared to law of neighboring India and China of up to 7 years of imprisonment, Nepalese law protecting health workers is less harsh [11]. Hence amending the law to make it stricter was one of NMA’s main demands. The medical community viewed Ordinance as historic expecting the violence to significantly decrease before a series of horrific events happened in September [10]. This brings up the following two issues: 1) Does toughening up penalty really work to stop these kinds of incidents? 2) Was it a "crime contagion"?

The violence against healthcare workers is multifaceted, with deep-rooted causes. First and foremost, the inadequate infrastructure and resources in Nepal's healthcare system have led to understaffed and overcrowded hospitals. A study found that 72.4% of doctors working in Kathmandu had burnout and 77.6% had secondary traumatic stress [12]. These elements can lead to significant delays and frustration among patients which can create a powder keg of discontent that can erupt into violence at any moment. Furthermore, misinformation and rumors regarding medical practices, especially in rural areas, can trigger violent outbursts. Accusations of malpractice are common following fatalities [ 13,14]. These occurrences are often fueled by media trials, which further create mistrust between healthcare providers and consumers.

The most significant factor is the lack of legal repercussions and swift actions for individuals who attack healthcare workers [11,14]. Likewise, the failure to prosecute professionals involved in medical negligence emboldens others to resort to violence when dissatisfied with medical services [13]. In most cases, the police themselves have not taken appropriate action against the attackers, further eroding healthcare workers’ faith in the system’s ability to protect them [ 11,15]. In most cases, the person involved in committing the violence is often connected with political parties [14]. For instance, the ward chairperson himself committed the crime in the Lamjung case [4]. The involvement of a third party in escalating the violence for financial gain is also very common [13,14,16]. For instance, in the Pokhara incident [5], a suspect who had been in police custody fifteen times also turned out to have been involved in the vandalism, and in Hetauda [2], the incident was carried out with the assistance of neighborhood thugs a month after the patient had passed away.

The psychological toll of such violence on healthcare workers is immeasurable. The constant fear of assault has led to widespread burnout, depression, and post-traumatic stress disorder [14]. They often work in a state of constant anxiety, which negatively impacts their performance and overall well-being. Doctors are reluctant to serve in remote areas and often hesitate to handle critical cases. 2318 doctors applied for Good Standing Certificate to go abroad according to Nepal Medical Council (NMC) compared to 954 doctors in 2022 [17]. NMC also claims that security issue is one of the factors contributing for a huge number of doctors leaving the country [18].

The escalating violence against healthcare workers in Nepal is a deeply concerning issue that demands immediate attention. It threatens not only the safety and well-being of healthcare professionals but also the functionality of the healthcare system as a whole [ 17,18]. The government should enforce already existing laws effectively to ensure a safe workplace, and at the same time, the process of filing malpractice complaints should be made easily accessible to the public to discourage them from resorting to violence [13,14].

## CRediT authorship contribution statement

**Leison Maharjan:** Conceptualization, Writing – original draft, Writing – review & editing.

## Declaration of competing interest

The authors declare that they have no known competing financial interests or personal relationships that could have appeared to influence the work reported in this paper.
